# Characterization of the complete mitochondrial genome of *Purpuricenus temminckii* (Coleoptera: Cerambycidae)

**DOI:** 10.1080/23802359.2019.1687357

**Published:** 2019-11-12

**Authors:** Wen-Jia Yang, Shu-Yan Yan, Kang-Kang Xu, Can Li

**Affiliations:** Guizhou Provincial Key Laboratory for Rare Animal and Economic Insect of the Mountainous Region, College of Biology and Environmental Engineering, Guiyang University, Guiyang, Guizhou, China

**Keywords:** *Purpuricenus temminckii*, Cerambycidae, mitogenome

## Abstract

The complete mitochondrial genome of *Purpuricenus temminckii* (GenBank accession number MN527358) is 15,689 bp in length, and contains 13 protein-coding genes, 22 transfer RNA genes (tRNAs), 2 ribosomal RNA genes, and a putative control region. The overall base composition is A (39.56%), C (17.52%), G (10.90%), and T (32.02%), with a high AT bias of 71.58%. All tRNAs have the typical cloverleaf structure except for *trnS_1_* and the length of them range from 62 to 71 bp. Phylogenetic tree showed that *P. temminckii* was clustered with three Cerambycidae species, which agree with the traditional classification.

The bamboo red longhorn beetle, *Purpuricenus temminckii* (Coleoptera: Cerambycidae), is a major forest pest and widely distributed in China, Korea, and Japan. The larvae of *P. temminckii* bore into the stem of bamboo, causing heavy damage to bamboo cultivation (Lim et al. 2014). In this study, adult specimens of *P. temminckii* were collected from Nanming District, Guiyang City, Guizhou Province, China (N26°33′, E106°47′), and deposited in the insect specimen room of Guiyang University with an accession number GYU-CoL-2019002.

The complete mitogenome of *P. temminckii* (GenBank accession number MN527358) is 15,689 bp in length with the typical insect complement of 13 protein-coding genes (PCGs), 22 transfer RNA genes (tRNAs), 2 ribosomal RNAs (*rrnL* and *rrnS*), and a putative control region (Boore 1999). The gene order and orientation of *P. temminckii* were identical to those observed in other coleopteran mitogenomes (Liu et al. 2014; Xu et al. 2017). Fourteen genes were transcribed on the minority strand (N-strand), whereas the others were oriented on the majority strand (J-strand). The overall base composition of *P. temminckii* mitogenome is A (39.56%), C (17.52%), G (10.90%), and T (32.02%), with a high AT bias of 71.58%. It is significant biased towards A + T with a positive AT-skew (+0.105) and negative GC-skew (−0.233).

The *P. temminckii* mitogenome harbors a total of 29 bp intergenic spacer sequences, which is made up of 8 regions in the range from 1 to 17 bp. The largest intergenic spacer sequence of 17 bp is located between *trnS_2_* and *nad1*. Gene overlaps were found at 14 gene junctions and involved a total of 41 bp, the longest 8 bp overlapping located between *trnY* and *cox1*. The length of 22 tRNAs varied between 62 bp (*trnC*) and 71 bp (*trnK* and *trnV*), comprising a total of 1448 bp. All tRNAs can be folded into the typical cloverleaf secondary structure, except for *trnS_1_* that the dihydrouridine arm forms a simple loop, which is similar to previous reports in other animal mitogenomes (Wolstenholme 1992; Yang et al. 2016). The *rrnL* is 1271 bp in length with A + T content of 74.67%, and the *rrnS* is 776 bp in length with A + T content of 70.49%. The 1058 bp control region is located between *rrnS* and *trnI*, and has a remarkably high A + T content (80.62%).

Eleven PCGs initiate with ATN as the start codons (ATG for *atp6*, *cox3*, *cob*, *nad4*, and *nad4L*; ATT for *atp8*, *nad2*, *nad3*, and *nad5*; ATC for *cox2*; ATA for *nad6*), however, cox*1* and *nad1* use ACC and TTG as start codons, respectively. Ten PCGs end with two types of complete stop codons, TAG (*atp8*, *cob*, *nad1*, and *nad3*) and TAA (*atp6*, *cox1*, *nad2*, *nad4*, *nad4L*, and *nad6*). The remaining PCGs ended with incomplete stop codon (T), including *cox2*, *cox3*, and *nad5*. Based on the concatenated amino acid sequences of 13 PCGs, the neighbor-joining method was used to construct the phylogenetic relationship of *P. temminckii* with 14 other representative beetles. The result showed that *P. temminckii* is clustered with three Cerambycidae species (Figure 1), which agree with the traditional classification.

**Figure 1.**
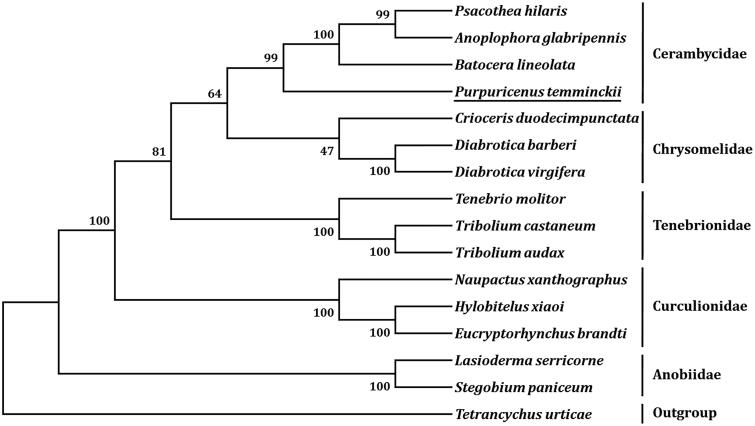
Phylogenetic tree showing the relationship between *P. temminckii* and 14 other beetles based on neighbor-joining method. GenBank accession numbers used in the study are as follows: *Anoplophora glabripennis* (NC_008221), *Batocera lineolata* (NC_022671), *Crioceris duodecimpunctata* (NC_003372), *Diabrotica barberi* (NC_022935), *Diabrotica virgifera* (KF658070), *Eucryptorhynchus brandti* (NC_025945), *Hylobitelus xiaoi* (NC_022680), *Lasioderma serricorne* (NC_038197), *Naupactus xanthographus* (KP306789), *Psacothea hilaris* (NC_013070), *Stegobium paniceum* (NC_036678), *Tetrancychus urticae* (EU345430), *Tenebrio molitor* (NC_024633), *Tribolium audax* (NC_024600), and *Tribolium castaneum* (NC_003081). *Tetrancychus urticae* was used as an outgroup. Beetle determined in this study is underlined.
